# Cure of Alzheimer’s Dementia in Many Patients by Using Intranasal Insulin to Augment an Inadequate Counter-Reaction, Edaravone to Scavenge ROS, and 1 or 2 Other Drugs to Address Affected Brain Cells

**DOI:** 10.3390/jcm12093151

**Published:** 2023-04-27

**Authors:** Jeffrey Fessel

**Affiliations:** Department of Medicine, University of California San Francisco, 2069 Filbert Street, San Francisco, CA 94123, USA; jeffreyfessel@gmail.com; Tel.: +1-415-563-0818

**Keywords:** Alzheimer’s dementia, cure, normal cognition, molecular events, changed functions, augment counter-regulation, brain cell-types, intranasal insulin, edaravone, pioglitazone, fluoxetine, lithium

## Abstract

The goal of treatment for Alzheimer’s dementia (AD) is the restoration of normal cognition. No drug regimen has ever achieved this. This article suggests that curing AD may be achieved by combination therapy as follows. First, with intranasal insulin to augment the body’s natural counter-reaction to the changes in brain cell-types that produced the dementia. Second, with edaravone to decrease free radicals, which are increased and causal in AD. Third, as described elsewhere, with one or two drugs from among pioglitazone, fluoxetine, and lithium, which address the brain cell-types whose changed functions cause the dementia. Insulin restores cerebral glucose, which is the main nutrient for brain neurons whose depletion is responsible for the dementia; and edaravone decreases ROS, which are intrinsic causes of neuropathology in AD. This combination of drugs is a potential cure for many patients with AD, and should be tested in a clinical trial.

## 1. Introduction and Background

### 1.1. Changed Functions of Brain Cell-Types Initiate Impaired Cognition; if Counter-Reactions to Those Changes Are Inadequate, Cognitive Loss May Worsen to the Point of Alzheimer’s Dementia (AD). Therefore, in Order to Cure AD, Inadequate Counter-Reactions Need Augmentation

This article presents the hypothesis that in order to cure AD, it is not only the changes that initiated the cognitive loss that should be addressed but, critically, the counter-reactions whose inadequacy enabled that loss to progress to dementia. The reasoning is based upon the fact that, just as in particle physics each particle has its anti-particle, the changed function of any biological mechanism in an organism induces a counter-reaction. In another article it was shown that the initiation of impaired cognition that leads to AD occurs from underactivity by astrocytes, oligodendrocytes, excitatory neurons, inhibitory neurons, and endothelial cells, and from overactivity by microglia [1]. Therefore, the perpetuation of cognitive loss that has already happened, and its worsening to the degree of dementia, must be due not only to the continuing presence of changed function in the brain cell-types that induced the loss but, critically, to an inadequate degree of the counter-reactions provoked by those changes. Had the counter-reactions been adequate, they would either have prevented the cognitive loss from starting or would have reversed it once it had begun. After dementia has developed, its reversal, i.e., cure might be achieved by augmenting the counter-reactionary responses.

In the above-mentioned article, combinations of two drugs (pioglitazone with either fluoxetine or lithium) were suggested as a way to address the changed functions in brain cells; the present article suggests that a combination of intranasal insulin plus edaravone is another way of augmenting what must be inadequate counter-regulatory responses. Combining both approaches offers a high chance for a potential cure of established AD.

### 1.2. The Benefit of Augmenting a Counter-Reaction

Published examples demonstrate that augmenting a counter-reaction may be therapeutically beneficial. The first couple of examples show benefit for patients. In the first of these, raising levels of T regulatory (Treg) type 1 (Tr1) cells decreased the occurrence of a graft-versus-host (GVH) reaction; the mechanism includes the secretion of IL-10 and TGF-β by Treg cells, which counter-regulate the aggressive T cells that are responsible for immunity and inflammation [2]; and at the site of inflammation, heat shock protein 60 (HSP60), for which Treg cells have receptors, may be part of a counter-regulatory feedback loop of the immune system [3]. A clinical trial involved 12 patients with advanced hematologic malignancies who had had their T-cells depleted and then received hematological stem cell infusions plus donor T lymphocytes pretreated with IL-10 (which promotes the formation of Tr1 cells) [4]. The goals were to improve immune reconstitution, and, via Tr1 cells, to decrease concomitantly and by counter-regulation the severity of a graft-versus-host (GVH) reaction. The four patients who survived experienced only moderate GVH reactions, and they were alive with complete disease remission and immunosuppression-free 7.2 years after the treatment. The second example involved 20 patients with refractory Crohn’s disease, for whom the intention was that Tr1 cells might counter-regulate and decrease the inflammatory reaction driving Crohn’s disease [5]. Tr1 cells were isolated from their peripheral blood, then the Tr1 cells were activated and administered intravenously. At weeks 5 and 8, 40% of the patients had a response, as shown by a reduction in the Crohn’s Disease Activity Index (CDAI) of ≥100 points. The third example has not yet involved patients, but shows that if counter-regulatory treatment to increase β-synuclein were available, it would be beneficial; that could be demonstrable in rodent models of AD, by using azacytidine. Aggregates of α-synuclein form Lewy bodies, and in patients with dementia and LB, the α-synuclein that forms the LB was almost absent when the cortical neurons contained β-synuclein [6], whereas in patients’ brains showing either LB pathology alone or LB pathology plus Alzheimer’s dementia all cortical areas showed a greatly diminished expression of β-synuclein [7]. 

This article will describe in detail the benefits from intranasal insulin and edaravone. Using insulin to counter-regulate inadequate levels of cerebral glucose is probably applicable to most causes of AD because glucose is the major cerebral nutrient but has poor cerebral stores. Edaravone scavenges free radicals and, thus, has a strong anti-oxidant effect in cellular systems [8]; for this reason, the use of edaravone is probably also applicable to most causes of AD, which all involve the accumulation of free radicals such as reactive oxygen species (ROS). ROS promote excessive lipid and protein peroxidation, DNA damage, and neuronal degeneration. Jia et al. demonstrated in a mouse-model of AD that was transgenic for APP/PS1 that edaravone significantly decreased brain levels of 3-nitrotyrosine by 27.5% in the neocortex, by 28.5% in the entorhinal cortex, and by 40.2% in the CA3; and that superoxide dismutase and glutathione peroxidase levels were significantly increased [9].

The combination of intranasal insulin and edaravone is potentially a way to cure AD in some percentage of cases. That is not 100%, despite that the two drugs probably provide benefits to most causes of AD because the underpinnings of AD are several and disparate—familial, deposition of amyloid and/or hyperphosphorylated tau, diabetes, cerebrovascular disease, possession of the *APOE* ε4 allele, trisomy 21, environmental toxins, etc.; and there might be unknown others with different underpinnings. Yet it would be a major step forward were insulin plus edaravone to cure even 20% of cases. The purpose of this article is to describe how each of these two drugs might provide general benefits for AD, and how each affects the specific brain cell-types whose changed functions affect the brain so as to cause AD. 

### 1.3. Brain Insulin and the Counter-Reactionary Responses to Reduced Cerebral Glucose Levels

It has been recognized for over 25 years that counter-regulation occurs in response to reduced cerebral glucose levels. For example, in 1997 Borg et al. studied the effect of a gradual reduction in plasma glucose using a hypoglycemic clamp in fasted rats [10]. Concurrent infusion into the ventromedial hypothalamus of 100 mM (but not 15 mM) D-glucose, reduced by ~85% the epinephrine, norepinephrine, and glucagon counterresponses to hypoglycemia. A level of cerebral glucose that is below a threshold leads to a corrective counter-reaction with increases of plasma levels of glucagon, epinephrine, norepinephrine, and growth hormone: De Feo et al. found that those thresholds occurred when plasma glucose was 72 mg/dL [11]. Gruetter et al. used magnetic resonance spectroscopy to assess cerebral levels in healthy persons whose plasma levels were raised to between ~72 and ~540 mg/dL [12]. They found that brain glucose is linearly correlated with plasma glucose (correlation coefficient 0.90) and that cerebral glucose is only ~25% of plasma levels; thus, with plasma levels between 72 and 125 mg/dL, the lower cerebral threshold level was ~18 mgs/dL and the cerebral upper-normal was ~31 mg/dL. Glucose is mainly stored as glycogen in astrocytes, and glycogen may be rapidly converted to energy when astrocytes degrade glycogen to lactate, which is released and taken up and consumed by neurons via the astrocyte-neuron lactate shuttle [13]. The percent of glycogen varies by brain region, and because it is a polymer, its absolute amount cannot be quantified. In the CA1, the percent stored in astrocytes is 78.2, 13 in microglia, and 8.7 in neurons, where its degradation is more immediately available (see Figure 3D in ref [14]). As regards AD, astrocytes numbers in the dentate gyrus were more reduced in Braak stages 3–4 than in stages 0–2 [15], and astrocytes in the hindbrain have a glucose sensor that works via the glucose transporter GLUT2, which activates calcium release from the ER that, in turn, activates the counter-regulatory responses [16].

The above counter-reactions to low glucose levels are important because the brain stores very little glucose [17,18,19]. In the modulation of intracellular, cerebral metabolic activity, insulin has a critical role by regulating signaling pathways, particularly in the hippocampus and hypothalamus [18,20]. One way is because the main transporters of glucose into neurons are GLUT3 and GLUT4, and they are insulin-dependent. In the normal brain, insulin controls neuronal uptake of glucose, so can compensate for cerebral levels of glucose that are below threshold [11]. Inadequate insulin-determined neuronal glucose is a risk factor for the occurrence of AD. That was seen by Baker et al., who studied non-seriously obese (body mass index = 27) subjects with pre-diabetes or diabetes and found that those with greater insulin resistance had an AD-like pattern of reduced cerebral metabolic rate of glucose in their frontal, temporal-parietal, and cingulate regions, that was independent of the subjects’ age, 2-h plasma glucose concentration, or APOE *ε4* allele status [21]; and their pre-diabetic or diabetic subjects recalled fewer items on the delayed memory test than their normal controls. Multiple reports using imaging by positron emission tomography (PET) with 2-[^18^F]fluoro-2-deoxy-D-glucose (FDG–PET) have shown that persons with familial and sporadic AD, as well as those with preclinical AD, have decreased cerebral glucose, particularly in the parietal and temporal cortices; they have also shown that declines in uptake have occurred at the times between normal cognition and mild cognitive impairment (MCI), and the times between MCI and dementia [22,23]. Ma et al. reported a meta-analysis of studies using FDG PET that included 93 patients that converted from amnestic, mild cognitive impairment (aMCI) to AD (age 57.8–77.7 years and MMSE scores 19.9–27.8) and 129 patients with aMCI who did not convert to AD (age 60–75.8 years and MMSE scores 23.9–28.3); the follow-up was 12 to 60 months [24]. Hypometabolism in the left posterior cingulate cortex (PCC)/precuneus predicted the conversion from aMCI to AD. 

Rodent studies confirm the importance of an adequate counter-reactionary response by insulin to cerebral hypoglycemia. Diggs-Andrews et al. tested the hypothesis that insulin acts directly in the brain to mediate the counterregulatory response to hypoglycemia. They subjected mice with knock-out of the neuron-specific insulin receptor to graded degrees of both hypoglycemic (100, 70, 50, and 30 mg/dL) and hyperinsulinemic (20 mU/kg/min) clamps, using the transcriptional marker *c-fos* to assess hypothalamic neuronal activation, and measuring regional brain glucose uptake by ^14^C-2deoxyglucose autoradiography [25]. They found that hypoglycemia caused a 3-fold reduction in the activation of neurons in the ventromedial hypothalamus and an impairment in the sympathoadrenal response. Further, insulin action was necessary for a full sympathoadrenal response to hypoglycemia. 

Thus, insulin determines an adequate neuronal uptake of glucose that is requisite for neuronal function, and an inadequate neuronal response to insulin is a risk factor for AD.

### 1.4. Intranasal Insulin, Cognition, and AD

Intranasal insulin has been shown to benefit cognition. Craft et al. reported studies in 104 patients with aMCI and 40 patients with mild or moderate AD [26]. Participants were treated with either 20 or 40 units of intranasal insulin for 4 months. As compared with placebo-assigned participants, those receiving both doses of insulin had significantly preserved Dementia Severity Rating Scale (DSRS) scores. Importantly, the groups using intranasal insulin showed reduced progression of hypometabolism in bilateral frontal, right temporal, bilateral occipital, and precuneus and/or cuneus regions during the 4-month treatment period. In a systematic review of eight studies with 328 participants, Shemesh et al. concluded that beneficial effects were induced by 160 IU/day of intranasal insulin [27]. Another systematic review by Avgerinos et al. involved 293 participants with MCI or AD; most studies reported that intranasal insulin administration produced improvement in verbal memory and in story recall, especially [28].

In brief, studies demonstrate that intranasal insulin benefits both AD and earlier forms of cognitive loss.

Nevertheless, although glucose reperfusion is a needed counter-reaction to cerebral hypoglycemia, activation of neuronal NADPH oxidase occurs during glucose reperfusion and may have unfavorable consequences by promoting ROS and neuronal death [29]. However, the use of edaravone significantly decreased levels of ROS that had been increased by high levels of glucose [30]. For this reason, the maintenance of cerebral glucose by insulin requires the concomitant use of edaravone to counter the possible formation of ROS.

### 1.5. Insulin and Astrocytes

Cai et al. demonstrated that astrocytes are a direct insulin target in the brain and that knock-out of the insulin receptor on astrocytes caused increased anxiety-like and depression-like behaviors in mice; they also showed that loss of insulin signaling in astrocytes impaired tyrosine phosphorylation of Munc18c, which leads to decreased exocytosis of ATP from astrocytes, which in turn causes decreased purinergic signaling on dopaminergic neurons [31]. Astrocyte numbers increased dose-dependently when Heni et al. added insulin to cultured human astrocytes [32]. It is also important that astrocytes have an important role in the pathogenesis of AD. Emphasizing their importance for cognition, astrocyte numbers in the dentate gyrus were more reduced in Braak stages 3–4 than in stages 0–2 [15]. Because astrocyte processes wrap around cerebral microvasculature, the morphological modifications of astrocytes affect micro-cerebral blood flow and, therefore, the nutrients available to neurons [33,34]; in addition, the processes of astrocytes contain aquaporin-4 (AQ4), which regulates water entry into endothelial cells [35]. The consequences of decreased numbers of astrocytes in AD include decreases in neural function and the passage of drugs into the brain.

### 1.6. Insulin and Oligodendrocytes

Oligodendrocytes have receptors for both insulin and insulin-like growth factor I (IGF-I). McMorris et al. cultured cells from rats’ brains and found that the number of oligodendrocytes was increased by exposure of the cultures to either insulin or IGF-1; IGF-1 was particularly effective, inducing a 60-fold rise in number of oligodendrocytes but a <2-fold increase in the number of non-oligodendroglial cells [36]. In this regard, IGF1 and IGF-2 both bind the insulin receptor [37].

### 1.7. Insulin and Synapses/Neurons

Pinelis et al. found that when cultures of rats’ neurons were exposed to glutamate, insulin protected them by preventing hyperactivation of the ionotropic N-methyl-D-aspartate receptors (NMDAR) and the deleterious consequences of a sustained increase in intracellular free calcium, synchronous mitochondrial depolarization, and increased intracellular superoxide anion radical (O_2_−^•^) production [38]. Mahmoud et al. reported that in neurons of mice, hypoglycemia up-regulated glutamate decarboxylase, causing more of the neuronally excitotoxic glutamate [39]. Kuboki et al. saw that insulin increased nitric oxide synthase (NOS) in endothelial cells, which potentially might cause an excess of free radical nitric oxide (NO) that would be scavenged by using edaravone [40]. Chiu et al. applied a range of light stimulation to living tadpoles, and made whole-cell recordings from tectal neurons [41]. Control tadpoles had wild type insulin receptors and others were transfected with dominant-negative insulin receptors that would reduce the efficacy of insulin; the latter tadpoles had significantly smaller light-evoked responses than controls, and as assessed by electron microscopy, their neurons had a reduced density of synapses.

In brief, the above results show the benefits that insulin provides to synapses and neurons.

### 1.8. Insulin and Endothelial Cells; Blood Flow

Insulin increases blood flow in peripheral tissues [42]. In the brain, Cranston et al. measured blood flow before and after insulin infusions, and found a small but significant increase in whole brain cerebral blood flow (CBF) (*p* = 0.04), but no difference in CBF between any brain region whether plasma insulin levels were low or high [43]. Of course, the levels of insulin in brain and plasma may be very different, but Akintola et al. assessed CBF with phase contrast MR-angiography and arterial spin labelling; the use of intranasal insulin as compared to placebo in older participants (mean age 65.2 years) increased perfusion through the occipital gray matter (65.2 mL/100 g/min vs. 61.2 mL/100 g/min, *p* = 0.001) and the thalamus (68.28 mL/100 g/min vs. 63.31 mL/100 g/min, *p* = 0.003); however, CBF did not show similar changes in younger participants (mean age 22.3 years) [44]. Novak et al. assessed CBF after intranasal insulin and saw an increase of perfusion that was greater in the insular cortex (*p* = 0.0003) in patients with diabetes as compared with controls [45]. 

### 1.9. Insulin, Endothelial Cells and the Dilemma of Nitric Oxide (NO)

Endothelial cells possess insulin receptors, and insulin stimulates them to produce nitric oxide synthase (eNOS) [40,46]. That poses a dilemma since NO, as a free radical, is deleterious, but is greatly beneficial as both a vasodilator and metabolic enhancer. While insulin stimulates the formation of NO by endothelial cells, it also inhibits the formation of NO by microglia [47]. Edaravone, if administered with insulin as suggested here, would scavenge NO and decrease release from endothelial cells of endothelin-1 (ET-1), which causes vasoconstriction [48]. Therefore, the net effect upon NO of administering both insulin and edavarone might be neutral. Further discussion is provided below, in the section ‘Explanation for a deleterious counter-reaction to elevated NO’.

### 1.10. Insulin and Microglia

Brabazon et al. exposed cultured microglia to the pro-inflammatory stimulus of lipopolysaccharide (LPS); administering insulin after LPS led to significantly reduced production of NO, ROS, and TNFα, and increased phagocytosis, showing that insulin reduced the pro-inflammatory M1 properties of microglia [47]. Haas et al. added insulin to microglia in culture, and showed an increase of phosphorylated Akt Ser473, which is an M2 microglial protein and reflects a switch from the pro-inflammatory M1 microglial isoform to the anti-inflammatory M2 isoform [49].

### 1.11. Beneficial Effect of Insulin in AD as Shown at the Level of Genes

Ravetti et al. analyzed a data set that showed the expression of 372 hippocampal genes in subjects with AD and controls [50]. The 372 genes were either up-regulated or down-regulated. For AD-relevant genes, those linked to synaptic function and neuronal plasticity had 21 down-regulated and 21 up-regulated; those linked to signaling of calcium had 14 down-regulated and 14 up-regulated; those linked to phosphatidylinositol (PI) had 7 down-regulated and 13 up-regulated; and for those linked to insulin, the data set had 6 genes down-regulated and 13 up-regulated. The major differences in the directions of change, whether upwards or downwards, may be interpreted as showing relatively unbalanced counter-reactions, whereas less well-defined differences indicate relatively balanced reactions. Does the relative up-regulation of the genes for PI and insulin reflect compensatory reactions whose decrement—if induced by therapy—would be deleterious, or to the contrary, does it indicate that such a decrement might be beneficial? In that regard, it is striking that in AD, PI (which translocates GLUT4 to the plasma membrane upon insulin receptor activation) was reduced by 40–50% in different areas of the brain cortex [51]; and some evidence also indicates that insulin is reduced in the AD brain; further, intranasal insulin has produced improved cognition [52]. Therefore, the best interpretation is that the relative increases of these genes are beneficially compensatory. In this regard, it was shown by Maffucci et al. that insulin specifically induced the formation of phosphatidylinositol in insulin-responsive cells [53].

### 1.12. Insulin also Benefits AD by Affecting the Extracellular Matrix (ECM)

It is in the ECM of the synaptic cleft where uptake of excitatory neurotransmitters, i.e., glutamate and aspartate occurs, and from which inadequate uptake causes excitotoxic neuronal death. The principal structural proteins in the ECM are collagen and elastin. Matrix metalloproteinases (MMP), particularly MMP-9, degrade the ECM to expedite uptake of glutamate and aspartate [54]. In this respect, insulin would be beneficial by augmenting the production of MMP-9 [55]. 

### 1.13. Edaravone Affects the Response to Free Radicals

The pharmacology of the FDA-approved drug edaravone, which is a scavenger of free radicals such as ROS, is described in a review by Begum et al. [56]. ROS include a variety of highly reactive free radicals, such as superoxide anion radicals (O_2_−^•^), hydroxyl radicals (−OH), per hydroxyl radicals (HO_2_*), singlet oxygen (^1^O_2_), hydrogen peroxide (H_2_O_2_), nitric oxide (NO·), and peroxynitrite (ONOO−).

Villa-Delfino et al. demonstrated that levels of ROS produced by granulocytes in response to a selective activator of PKC, which promotes the formation of NADPH oxidase (NOX) [30,57], were decreased by up to 8-fold by edaravone [30]. Treatment with edaravone also significantly ameliorated the clinical severity of experimental autoimmune encephalomyelitis (EAE), and this was accompanied by a reduction in the formation of NOS and, therefore, of the free radical NO− [58]. Various enzyme systems may produce ROS, including NOX, which transfers electrons from NADPH to molecular oxygen to generate O_2_−^•^ and/or H_2_O_2_. The resulting oxidative stress produces damage to lipids, proteins, and DNA, followed by cell death. Besides edaravone, antioxidant enzymes such as superoxide dismutase (SOD), glutathione peroxidase (GPx), and catalase also counter ROS, but unlike endaravone, they are unavailable for clinical use. NOX ligand receptors activate pathways that are responsible for caspase formation and the secretion of pro-inflammatory cytokines, such as IL-1β, IL-6, and IL-18. Edaravone counteracts those deleterious consequences of activated NOX ligand receptors, and scores of reports document the benefit to cognition from this drug when it has been administered to rodent models that develop impaired cognition. Among the NOX isoenzymes, NOX1 is found in astrocytes, neurons, and microglia; NOX2 is found in neurons and microglia [59].

### 1.14. Edaravone and Oligodendrocytes

Agresti et al. reported that edaravone increased the expression of genes for myelin basic protein (MBP) by almost 6-fold in oligodendrocyte precursor cells (OPC), which is highly beneficial for myelination of axons; and it significantly increased formation of MBP RNA by cultured cerebellar slices [59]. Takase et al. saw that following cerebral hypoperfusion in rats, edaravone prevented both the oligodendrocyte damage and the consequent white matter lesions, and the cognitive deficits [60]. The oligodendrocyte damage was presumably from oxidative stress due to free radicals, because in another series of experiments, Takase et al. demonstrated that OPCs exposed to H_2_O_2_ had significantly less (*p* < 0.05) death when protected by edaravone [61].

Cerebral hypoperfusion in rats caused decreases in levels of the synaptic proteins PSD93, PSD95, and NR2A/B; those decrements were lessened by the administration of edaravone (*p* < 0.01) [62]. Jiao et al. reported that edaravone reduced amyloid β (Aβ) deposition in the APPswe/PS1 mouse model of AD, alleviated the oxidative stress induced by Aβ, and attenuated tau hyperphosphorylation, glial activation, neuroinflammation, neuronal loss, and synaptic dysfunction; and in cultures of cortical neurons, they found that edaravone prevented neurite collapse and cell death—all of these effects of edaravone are highly beneficial [25].

### 1.15. Edaravone, Endothelial Cells/Pericytes, and Endothelin-1: Another Dilemma

ROS that was induced by Aβ provoked the release of endothelin-1 (ET-1) and its ligation by pericyte ET-1 receptors, which activated pathways leading to the constriction of brain capillaries [63]. The beneficial effect of edaravone with respect to ET-1 released by cerebral endothelial cells has been extensively studied in China, with scores of reports, just one of which will be mentioned for its unusual interest. That report was by Zhou et al., who studied 108 patients with cerebral infarction; all received conventional treatment, but half of the patients also received edaravone 30 mg bid for 14 days [48]. At baseline, levels of ET-1 and NO, and the MMSE and MocA scores were almost identical and non-significantly different in the two groups; but after treatment, ET-1 was 33.9% lower and NO was 50.2% higher in the group that received edaravone, and in that group the MMSE scores were 8.3% higher and the MocA scores were 6.8% higher than in the control group that did not receive edaravone (*p* < 0.001 for all differences). Similar results with edaravone significantly decreasing ET-1 levels were reported by Li et al. and by Yin et al. [64,65]. Edaravone has also been widely used in Japan. Ishibashi et al. reported 625 consecutive patients seen over a 14-years period who were admitted to their institution within 48 h after onset of acute ischemic stroke; of the 625 patients, 237 (37.0%) received both edaravone and the conventional treatment, (*n* = 237); the rest received the conventional treatment only [66]. Those receiving treatment with edaravone had less death compared to the conventional treatment (*p* = 0.099 after adjustments for age and gender). In brief, although none of the studies described above were randomized, the overall data suggest that edaravone—via reducing the effect of ET-1 released by endothelial cells—may provide favorable clinical results. Nevertheless, there is a dilemma, discussed in detail below, in that the patients who received edaravone developed decreased ET-1 but increased NO levels; this result was beneficial vis a vis increased cerebral microvascular dilatation, but deleterious vis a vis the increase in free radicals [66]. As mentioned above, the recipients of edaravone developed higher MMSE scores [66]; thus, despite the increased NO, the end result from using edaravone in AD would be beneficial.

### 1.16. Edaravone and Microglia

Edaravone inhibited the microglial activation induced by LPS, and shifted the microglial phenotype from M1 (pro-inflammatory) to M2 (anti-inflammatory) [67].

### 1.17. Explanation for a Deleterious Counter-Reaction to Elevated NO

Strengthening the counter-reactions runs the risk that some are beneficial but some may be deleterious. Using intranasal insulin and edaravone in order to counter the reduced endothelial cell activity in AD generates free radical NO, which is a potentially deleterious counter-reaction. An apparent mystery concerning high levels NO is exemplified by the findings from a study reported by Zhou et al. (see above) in which half of the patients received edaravone [48]. Although baseline levels of NO and the MMSE and MocA scores were almost identical in the two groups, after treatment, NO was 50.2% higher, and the MMSE and MocA scores were higher by 8.3% and 6.8%, respectively, in those who received edaravone. If NO was so much higher and deleterious, then why were the cognitive test scores so significantly improved when they would be expected to have been a lot worse from the increased NO? A further question is, why were the NO levels high when they might be expected to have been scavenged by edaravone? Explanations are as follows.

First, why were the cognitive test scores so significantly improved in the presence of high NO? There are three NOS genes, neuronal (nNOS), inducible (iNOS), and endothelial (eNOS). A critical element regarding the disposition of NO is the duration of its production, which varies according to the NOS isotype: production by nNOS and eNOS has a duration of minutes or hours, but for iNOS it is days [68]. Since NO is highly diffusible and quickly enters erythrocytes for storage, NO resulting from a brief duration of its production by eNOS or nNOS becomes stored so that a high initial, local concentration quickly declines to a low level; but NO resulting from a prolonged, high production by iNOS saturates the storage in erythrocytes, and the continued production of NO produces a high level. Importantly, the bioavailability of NO becomes decreased because it interacts with O_2_−^•^ to form peroxynitrite (ONOO−). Although peroxynitrite would impair cognition, that would not affect the patients reported by Zhou et al. because it would have been scavenged by the edaravone; and, in addition, any residual NO would also have been scavenged [48]. That is one reason why the patients reported by Zhou et al. [48] had improved cognition in the face of high NO levels. 

Second, why were NO levels high despite evaravone? This question is answered by data from Kawasaki et al., who created cerebral ischemia in mice by occluding both common carotid arteries with clips, then produced reperfusion by removing the clips [69]. Three hours after reperfusion, nNOS levels were 33% lower in the mice treated with edaravone than in controls; but total NOS levels were higher by 27% (NS) and 33% (*p* < 0.05) in the mice treated with edaravone than in controls. Kawasaki et al. speculated that edaravone scavenged the free radicals, protected the endothelial cells, promoted eNOS activity, and increased NO production. Presumably, by removing free radicals, edaravone would affect depolarization, accounting for the findings of Otani et al. that long term potentiation (LTP) increased in the rat hippocampus after edaravone administration [70].

In brief, the data reported by Zhou et al. [48] are explicable.

## 2. Discussion

The goal of therapy for AD is the cure of dementia, i.e., the restoration of cognition that is normal for the person’s age and gender. Anything less is inadequate. It must be recognized, however, that no single therapeutic formula is likely to cure all cases, because the underpinnings of AD are several and disparate—familial, deposition of amyloid and/or hyperphosphorylated tau protein, diabetes, cerebrovascular disease, possession of the APOE ɛ4 allele, trisomy 21 with three copies of the gene for Aβ, environmental toxins, etc. If successful treatment for an individual case of AD depended upon the cause of AD in that patient, and if there were five separate causes of phenotypic AD with each of those five causes requiring its particular pharmacotherapy, then the likelihood that one of the five pharmacotherapies is correct for an individual case would depend on the percentage distribution of the five causes within the AD population from which a particular patient derives. On the one hand, if each of the five causes is equally likely, then the probability that one of the five pharmacotherapies is correct for a given patient would be 20%. If there were six causes requiring six separate pharmacotherapies, then the probability of one of them being correct would be 16%, etc. On the other hand, that calculation assumes that the causes are equally distributed within the population. If they were unequally distributed, then the likelihood of a particular solution being correct for an individual patient would be imponderable.

Imponderable also is the percentage of a population with AD that might be cured by a combination of intranasal insulin, edavarone, plus pioglitazone with either fluoxetine or lithium. However, evidence presented above suggests that it might cure a substantial percentage of patients, because the drugs address glucose, which is the major nutrient for the brain, ROS that are increased in the majority of cases of AD, and the brain cells whose changed functions are responsible for the dementia. It would be an indisputable benefit to patients and society, and a major step forward, were insulin and edaravone to cure only 20% of cases. It is hoped that the evidence adduced in this article supports the hypothesis that the suggested treatment might achieve at least that goal. 

A previous article suggested that curing AD requires addressing the changes in function of the brain cell-types that determine the neuropathology that produces the dementia [1]. The merit of that approach lies in the fact that 10 available drugs benefit the changed functions of those brain cell-types. Interestingly, that approach is not at odds with the hypothesis suggested in the present article, because the brain cell-types with changed functions that determine AD are astrocytes, oligodendrocytes, neurons, endothelial cells, and microglia; as shown above, insulin and edaravone also benefit all of those brain cell-types. It is the basis for addressing the brain cell-types that differentiates the two approaches: one uses medications that address the changed functions, whereas the other strengthens the counter-reactions to those changed functions. The regimens suggested for two drugs were pioglitazone plus either fluoxetine or lithium. Therefore, combining either one or two of those three drugs, and administering them with intranasal insulin plus edaravone, might offer the best chance to cure many patients with Alzheimer’s dementia. 

A clinical trial is advocated in order to demonstrate both the validity of benefit from intranasal insulin plus oral edaravone, and the safety of the regimen. It is emphasized that administration of these two drugs would be an off-label use.

## 3. Conclusions and Summary

- The goal of treatment for Alzheimer’s dementia (AD) is a cure, i.e., the restoration of normal cognition;
- Curing AD may be achieved by augmenting the body’s natural counter-reactions to the changed functions of brain cell-types that produced the dementia;
- Intranasal insulin plus edaravone would augment most of the counter-reactions to the changed functions of brain cell-types that cause Alzheimer’s dementia, potentially curing many cases;
- The addition of one or two drugs from among pioglitazone, fluoxetine, and lithium would address the affected brain cell-types and fortify the efficacy of intranasal insulin plus edaravone;
- The suggested regimen is likely to be curative for many individuals afflicted by AD, although no single therapeutic formula is likely to cure all cases because the clinical manifestations of AD have several underlying causes.

## Data Availability

Not applicable.
